# Overexpression of SerpinE2/protease nexin-1 Contribute to Pathological Cardiac Fibrosis via increasing Collagen Deposition

**DOI:** 10.1038/srep37635

**Published:** 2016-11-23

**Authors:** Xuelian Li, Dandan Zhao, Zhenfeng Guo, Tianshi Li, Muge Qili, Bozhi Xu, Ming Qian, Haihai Liang, Xiaoqiang E, Samuel Chege Gitau, Lu Wang, Longtao Huangfu, Qiuxia Wu, Chaoqian Xu, Hongli Shan

**Affiliations:** 1 Department of Pharmacology (State-Province Key Laboratories of Biomedicine-Pharmaceutics of China, Key Laboratory of Cardiovascular Research, Ministry of Education), College of Pharmacy, Harbin Medical University, Harbin, China; 2 The second Clinical Medical School of Inner Mongolia University for Nationalities, Inner Mongolia Forestry General Hospital, Inner Mongolia, China; 3 Department of Orthopaedics, the First Affiliated Hospital, Harbin Medical University, Harbin, China; 4 Department of Pharmacy and Complementary Medicine, School of Health Sciences, Kenyatta University, Nairobi, Kenya

## Abstract

Although increases in cardiovascular load (pressure overload) are known to elicit ventricular remodeling including cardiomyocyte hypertrophy and interstitial fibrosis, the molecular mechanisms of pressure overload or AngII -induced cardiac interstitial fibrosis remain elusive. In this study, serpinE2/protease nexin-1 was over-expressed in a cardiac fibrosis model induced by pressure-overloaded via transverse aortic constriction (TAC) in mouse. Knockdown of serpinE2 attenuates cardiac fibrosis in a mouse model of TAC. At meantime, the results showed that serpinE2 significantly were increased with collagen accumulations induced by AngII or TGF-β stimulation *in vitro*. Intriguingly, extracellular collagen in myocardial fibroblast was reduced by knockdown of serpinE2 compared with the control *in vitro*. In stark contrast, the addition of exogenous PN-1 up-regulated the content of collagen in myocardial fibroblast. The MEK1/2- ERK1/2 signaling probably promoted the expression of serpinE2 via transcription factors Elk1 in myocardial fibroblast. In conclusion, stress-induced the ERK1/2 signaling pathway activation up-regulated serpinE2 expression, consequently led accumulation of collagen protein, and contributed to cardiac fibrosis.

Myocardial fibrosis is an important pathophysiological process of excessive collagen deposition and are associated with myocardial stiffness and cardiac systolic and diastolic dysfunction. Along with the proliferation of cardiac fibroblasts, extracellular matrix proteins are excesssively synthesized and accumulated leading to a mass production of collagen^1^,^2^, and other substances^3^. Since the complexity of the mechanism underlying myocardial fibrosis, there is an increasing interests in the seeking novel mechanisms and new biomarkers regarding cardiac fibrosis.

SerpinE2 (Serpin Peptidase Inhibitor, Clade E, Member 2) is a 44 kDa to 50 kDa glycoprotein that is encoded by the SERPINE2 gene on human chromosome 2q99-q35^4^, which is also known as Protease Nexin-1 (PN-1), Glia-Derived Nexin (GDN), or PI-7 etc. SerpinE2/PN-1 expression is observed in a variety of cell types^5^,^6^, such as fibroblasts, vascular smooth muscle cells^7^, endothelial cells^8^, platelet particles^9^,^10^, the tumor cells, and so on. SerpinE2/PN-1 is secreted by fibroblasts^11^, and several other cultured cells as well^5^, which inhibits serine proteases including thrombin, urokinase, urokinase-type plasminogen activator (u-PA)^12^, tissue-type plasminogen activator (t-PA)^13^, plasmin, and trypsin^14^.

From phylogenetical point of view, serpinE2 is the closest relative of plasminogen activator inhibitor type 1(PAI-1)^15^, also as known SerpinE1. A recent observation has shown that PAI-1 is tightly linked with tissue fibrosis including all important organs such as the heart, lung, kidney, and liver, even the skin^16^. Therefore, we have a strong reason to believe that that serpinE2 may play a similar role in pathophysiological process of cardiac fibrosis. In order to test our hypothesis, we selected the TAC model and cultured mycardial fibroblast that were treated with angiotensin (ANG-II) and tissue growth factor-β (TGF-β), respectively, to elucidate the potential effects of serpinE2 on cardiac fibrosis and related cell signaling. These results have demonstrated for the first time that serpinE2 is one of key players in pathophysiological process of cardiac fibrosis and a potential target for clinical management of cardiac fibrosis and future pharmacological convention.

## Results

### Serpin peptidase inhibitor clade E member 2 (SerpinE2) expression increases in pathological cardiac fibrosis ***in vivo***

**Figure 1:**
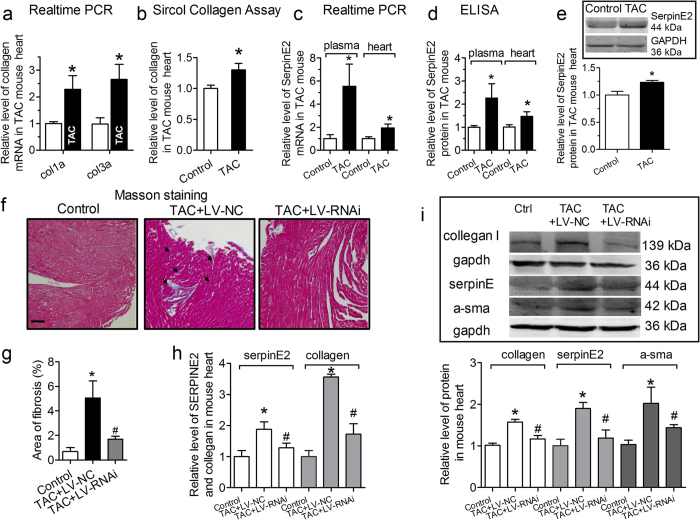
The expression of SerpinE2 increases in transverse aortic constriction (TAC)-induced mouse cardiac myocardial fibrosis model. (**a**) Collagen I/III (col1a/col3a) mRNA levels was detected by qRT-PCR. *n* = 8. *P < 0.05 vs. control. (**b**) Sircol™ Collagen Assay was used to quantify the total collagen contents in myocardial tissue from TAC models. *n* = 10. *P < 0.05 vs. control. (**c**) The expression of serpinE2 mRNA in plasma (*n* = 5, *P < 0.05 vs. control) and myocardium (*n* = 8, *P < 0.05 vs. control) of TAC mice using qRT-PCR. (**d**) The level of serpinE2 protein in plasma (*n* = 7, *P < 0.05 vs. control) and myocardium (*n* = 10, *P < 0.01 vs. control) of TAC mice using ELISA. (**e**) Western blot analysis of serpinE2 protein and statistical analysis (*n* = 6, *P < 0.01 vs. control). Full-length blots/gels are presented in Supplementary Figure 1. (**f**) Representative sections of heart with Masson staining. The fibrotic tissues are stained as blue, as indicated by the arrows. Scale bars: 200 μm. (**g**) Collagen deposition was quantified by automated image analysis and expressed as percentage of tissue area. *n* = 6. (**h**) The relative protein level of serpinE2 and collagen was detected by ELISA and Sircol™ Collagen Assay in Knockdown -serpinE2 mice. *n* = 6. **(i)** The protein of collagen, serpinE2 and α-sma were decreased in Knockdown-serpinE2 mice. Full-length blots/gels are presented in Supplementary Figure 2 and Supplementary Figure 3 (*n* = 6, ^#^*P* < 0.05 vs. TAC + LV-NC).

It has well been accepted that surgical transverse aortic constriction (TAC) induces cardiac fibrosis after 4 weeks^17^,^18^. As shown in Fig. 1a,b,f, the marked collagen deposition were observed in TAC group, suggesting dramatic fibrosis along with pathphysiological process of cardiac hypertrophy in TAC mice at 4 weeks. Approximately 85% of total myocardial collagen is type I, and 11% is collagen type III in the heart, which typically form thin fibers and maintain the elasticity of the matrix network^3^,^19^ in the physiological condition. Therefore, we have a reason to believe that TAC would cause cardiac fibrosis. To test this hypothesis, Collagen types I (col1a) and III mRNA (col3a) were evaluated by RT-PCR. The expression of col1a and col3a mRNA were increased in the interstitial tissue of TAC mouse (Fig. 1a). As expected, total collagen content was increased significantly by about 1.30 fold detected by Sircol™ Collagen Assay Kit (Fig. 1b).

To find out the connection of serpinE2 with cardiac fibrosis, the expression for serpinE2 mRNA were conducted using qRT-PCR and these results showed that the expression of serpinE2 mRNA were obviously increased ~5.54 and ~1.94 fold in TAC mouse plasma and in mouse myocardium, respectively (Fig. 1c). Then the protein expressions of serpinE2 were detected by ELISA assay and the results showed that serpinE2 protein expression was significantly increased ~2.35 fold in plasma of TAC mice compared with control mice (Fig. 1d), which was consistent well with the notion of ELISA and western blot showing ~1.29 fold (Fig. 1d) and ~1.23 fold (Fig. 1e) increases in myocardium of TAC mice. SerpinE2-RNAi-lentivirus were injected via the tail vein of mouse. One week after injection, these mice were subjected to pressure overload by TAC four weeks as TAC + LV-RNAi group (TAC + lentivirus -serpinE2-RNAi). The interstitial fibrotic area of TAC-LV-NC mouse (TAC + lentivirus-Negative control) increased obviously compared with control animals (0.69 ± 0.66% for control vs. 4.95 ± 2.92 for TAC-LV-NC, P < 0.05) (Fig. 1g). The interstitial fibrotic area of TAC + LV-RNAi was reduced compared with TAC-LV-NC in mouse heart (1.70 ± 0.49 for TAC + LV -RNAi, *P *< 0.05). To further confirm the serpinE2 was took part in the progress of cardiac fibrosis, expression of serpinE2 and collagen were detected *in vivo*. SerpinE2-RNAi-lentivirus induced an obviously decreased in serpinE2 level *in vivo*, moreover, knock-down serpinE2 also inhibited the content of collagen compared with TAC-LV-NC group (Fig. 1h). Western blotting was used to examine the protein expression levels of collagen I (Col1), alpha smooth muscle actin (α-SMA), and serpinE2. The expression of α-SMA, col1 and serpinE2 in TAC mouse of knock-down serpinE2 were all suppressed compared with TAC-LV-NC group (Fig. 1i).

### ANG-II-induced fibrosis and mediated increase in serpinE2 expression

**Figure 2:**
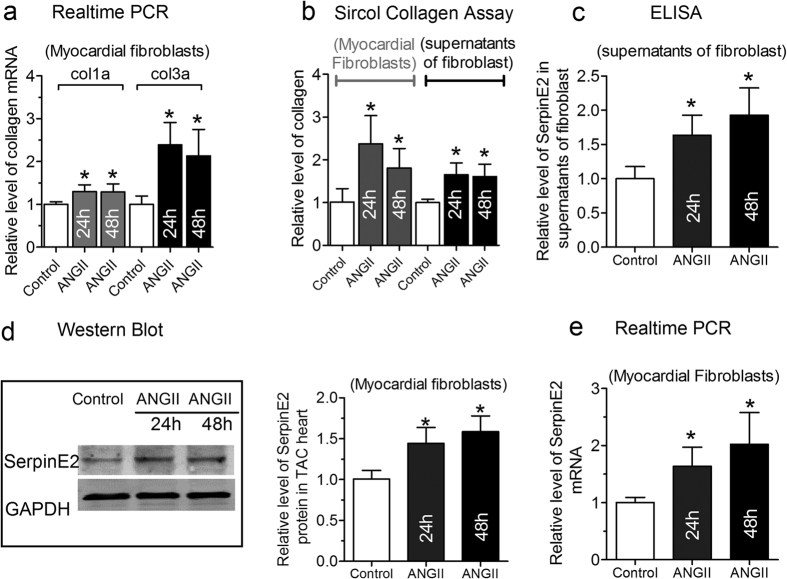
ANG-II-mediated increase in cardiac fibrosis and the serpinE2 expression. (**a**) qRT-PCR assay was applied for the detection of collagen I and collagen III mRNA expression in myocardial fibroblasts (*n* = 6, *P < 0.05 vs. control) in 50 nM AngII induced collagen deposition; (**b**) Sircol™ Collagen Assay was used to quantify the total collagen concentration both in fibroblasts and in the supernatants of fibroblast induced by AngII (*n* = 6, *P < 0.05 vs. control); (**c**) The expression of serpinE2 was detected by ELISA in the supernatants of fibroblasts (*n* = 6, *P < 0.05 vs. control); (**d**) Western blot analysis of serpinE2 protein (left panel) and statistical analysis (right panel) (*n* = 6, *P < 0.01 vs. control). Full-length blots/gels are presented in Supplementary Figure 4; (**e**) qRT-PCR was also used to detect expression of serpinE2 mRNA in myocardial fibroblasts stimulated by AngII (*n* = 8, *P < 0.01 vs. control).

AngII stimulation is linked to cardiac remodeling characterized by fibrosis with collagen accumulation. Consistently, as the result, the mRNA level of Collagen I and III were obviously increased in myocardial fibroblast after 24 h and 48 h treatment with 50 nM AngII and compared with the control group (Fig. 2a). Total contents of collagen were increased in fibroblast and in the supernatants of fibroblast (Fig. 2b), suggesting that ANG-II-mediated the secretion of collagen from fibroblasts into ECM. According to database of UniProtKB and Swiss-Prot for SERPINE2 Gene, serpinE2 is a kind of protein that is more likely to be secreted into the extracellular space, rather than or less into cytosol. Therefore, we have a reason to believe that the serpinE2 may play a certain function at the cell membrane and it can could be detected in the culture medium^10^,^11^. Based upon this regard, we then detected serpinE2 content in the supernatants of myocardial fibroblasts by ELISA assay. Compared with the control group, serpinE2 level was significantly increased about 1.63-fold and 1.92-fold, respectively, after 24 h and 48 h treatment with 50 nM AngII (Fig. 2c). It means that AngII very likely to promote serpinE2 secretion from fibroblasts into the supernatants. The data from western blot and qRT-PCR also showed a corresponding increment of serpinE2 in myocardial fibroblasts (Fig. 2d and e).

### TGF-β-mediated fibrosis and increase in serpinE2 expression

**Figure 3:**
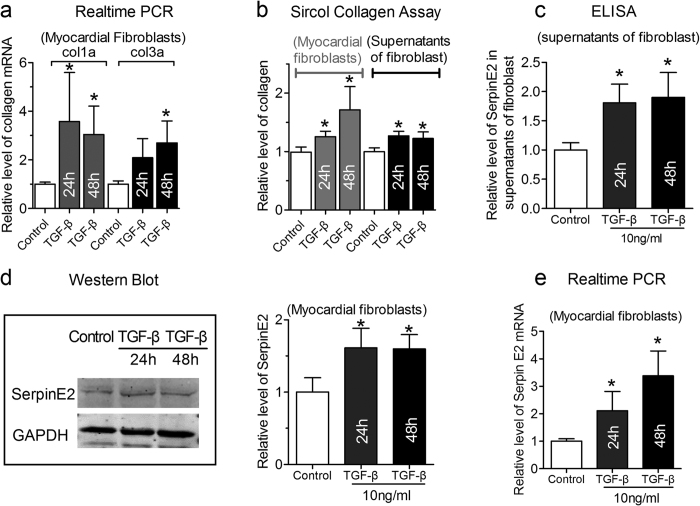
TGF-β-induced fibrosis and mediated increase in serpinE2 expression. (**a**) qRT-PCR assay was applied for detection of collagen I and collagen III mRNA expression in myocardial fibroblast (*n* = 6, *P < 0.05 vs. control) in TGF-β-induced fibrosis; (**b**) Sircol™ Collagen Assay was used to quantify the total collagen concentration both in fibroblasts and in the supernatants of fibroblasts (*n* = 6, *P < 0.05 vs. control); (**c**) The expression of serpinE2 was detected by ELISA in the supernatants of fibroblast (*n* = 6, *P < 0.05 vs. control); **(d**). Western blot analysis of serpinE2 protein (left panel) and statistical analysis (right panel) (*n* = 6, *P < 0.01 vs. control). Full-length blots/gels are presented in Supplementary Figure 5; **(e)**. qRT-PCR was also used to detect expression of serpinE2 mRNA in myocardial fibroblasts stimulated by TGF-β (*n* = 8, *P < 0.01 vs. control).

It has well been accepted that the activation of renin-angiotensin-aldosterone system would increase levels of active TGF-β that plays an important function in pressure overload-induced cardiac fibrosis^20^ and lead to increase in the mRNA expressions of collagen I and III in myocardial fibroblasts^20^,^21^. Compared with the control group, the total collagen concentration detected by Sircol™ Collagen Assay were significantly elevated in myocardial fibroblasts after 24 h and 48 h stimulation with TGF-β 10 ng/ml (Fig. 3a and b), meanwhile, quantified serpinE2 secretion by ELISA assay into the supernatants of fibroblast culture medium showed accordant changes with collagen content. These results indicate that TGF-β also promotes serpinE2 secretion from fibroblasts into the supernatants (Fig. 3c). The supportive results were accordingly also obtained using western blot and qRT-PCR showing the up-regulation of both protein and mRNA expression of serpinE2 in myocardial fibroblasts treated with TGF-β (Fig. 3d and e).

### SerpinE2 is expressed mainly in myocardial fibroblast and exogenous serpinE2 increased the content of collagen

**Figure 4:**
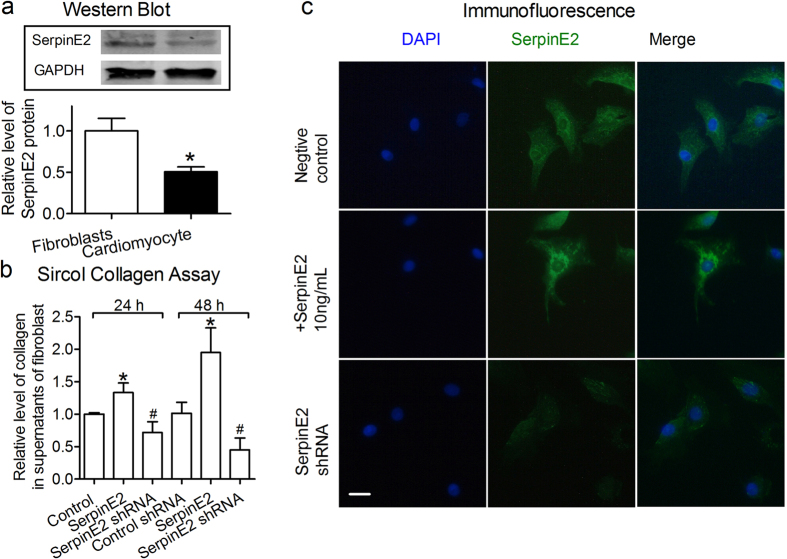
The changes of collagen content induced by exogenous serpinE2. (**a**) Differential expression of serpinE2 in myocardial fibroblast and myocardial cell. *n* = 3. *P < 0.05 vs. control. Full-length blots/gels are presented in Supplementary Figure 6. (**b**) Relative level of collagen in supernatants of fibroblast by over-expression of serpinE2 or knock-down of serpinE2. *n* = 6. *P < 0.05 vs. control, ^#^*P* < 0.05 vs. serpinE2 group. (**c**) The Immunofluorescence for serpinE2 in myocardial fibroblast. Scale bar:10 μm.

The histological structure of the heart is mainly composed of mast myocardial cells and myocardial fibroblasts. SerpinE2 is both expressed in myocardial fibroblast and myocardial cell as well, moreover, the expression of serpinE2 in the myocardial fibroblast was higher than that in myocardial cell (Fig. 4a). Subcellular locations from UniProtKB/Swiss-Prot database for SERPINE2 Gene showed that serpinE2 is mostly secreted into extracellular space, next is in cytosol. Since serpinE2 can be secreted by fibroblasts into extracellular fluid^14^, the exogenous serpinE2 (serpinE2-gst Fusion Protein) was added to cardiac fibroblasts at final concentration of 10 ng/ml for 24 h and 48 h. Then the level of collagen were all increased significantly about 1.33-fold and 1.95-fold compared with the control in supernatant of fibroblasts detected by Sircol™ Collagen Assay at 24 h and 48 h (Fig. 4b). On the contrary, when cardiac fibroblasts was knock-down of serpinE2 with serpinE2 shRNA for 24 h and 48 h, the content of collagen was obviously reduced compared with treated with serpinE2 group (Fig. 4b). The immunofluorescence showed the expression of serpinE2, when the exogenous serpinE2 was added or knock-down of serpinE2 in myocardial fibroblast. The expression of serpinE2 is increased in fibroblasts treated with exogenous serpinE2 than that in the control (Fig. 4c). It illustrated the exogenous serpinE2 can entry into the fibroblasts by some way. In addition it was supported by the hypothesis that serpinE2/ protease nexin-1 can induce endocytosis binding to uPA/uPAR complexes mediated by LRP^22^,^23^.

### Knock-down of serpinE2 reduces the collagen deposition in supernatant of fibroblasts

**Figure 5:**
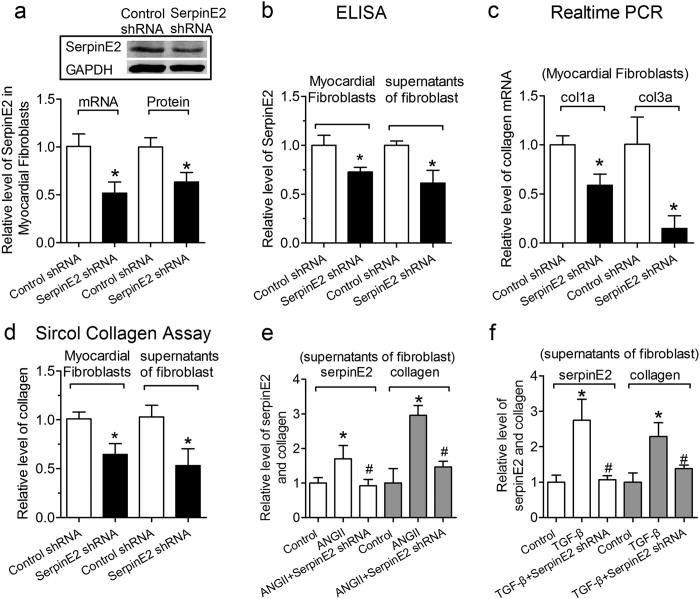
Knock-down of serpinE2 reduces the collagen deposition in supernatant of fibroblasts. (**a**) After 24 h transfection with serpinE2 shRNA, the mRNA level of serpinE2 was detected by qRT-PCR in cardiac fibroblasts. *n* = 10, *P < 0.05 vs. control; the protein level of serpinE2 was detected by western blot in preparation. *n* = 6, *P < 0.05 vs. control. Full-length blots/gels are presented in Supplementary Figure 7. (**b**) After transfection with serpinE2 shRNA, the protein level of serpinE2 was also detected by ELISA assay both in the fibroblast and the supernatants. *n* = 6, *P < 0.05 vs. control. (**c**) The mRNA expression for collagen was measured by qRT-PCR after transfection with serpinE2 shRNA. *n* = 10, *P < 0.05 vs. control. (**d**) Collagen expression were also measured by Sircol™ Collagen Assay both in the fibroblasts and the supernatants. *n* = 7, *P < 0.05 vs. control. (**e**) Knock-down of serpinE2 can inhibit the collagen deposition induced by ANG-II. *n* = 6. *P < 0.05 vs. control, ^#^*P* < 0.05 vs. AngII group. **(f)** Knock-down of serpinE2 suppressed the collagen deposition induced by TGF-β. *n* = 6. *P < 0.05 vs. control, ^#^*P* < 0.05 vs. TGF-β group.

After 24 h transfection of serpinE2 shRNA, the level of serpinE2 mRNA was decreased by 51% compared with the control shRNA (Fig. 5a), which was consistent with the data from western blot showing the knockdown of the serpinE2 protein by 85% (Fig. 5a), and serpinE2 by 72% detected by Elisa in myocardial fibroblast (Fig. 5b). Moreover, a similar trend of changes in serpinE2 released in the supernatant was also verified 61% (Fig. 5b). Compared with the control group, either protein or mRNA expression for collagen was down-regulated (Fig. 5c) by knock-down of serpinE2. The total collagen was paralleled lowered in cardiac fibroblasts and supernatant of fibroblasts, respectively, under the same condition (Fig. 5d). The exposure of cardiac fibroblasts to Ang II (50 nM) caused a significant up-regulation of serpinE2 and collagen production (P < 0.05), which was attenuated by knock-down of serpinE2 with serpinE2 shRNA of 50 nM respectively (Fig. 5e). TGF-β promotes secretion of serpinE2 and collagen from fibroblasts into the supernatants (Fig. 5f). knock-down of serpinE2 with serpinE2 shRNA significantly inhibited the collagen secretion induced by TGF-β (Fig. 5f).

### ERK1/2 signal pathway involved in serpinE2-mediated collagen deposition

**Figure 6:**
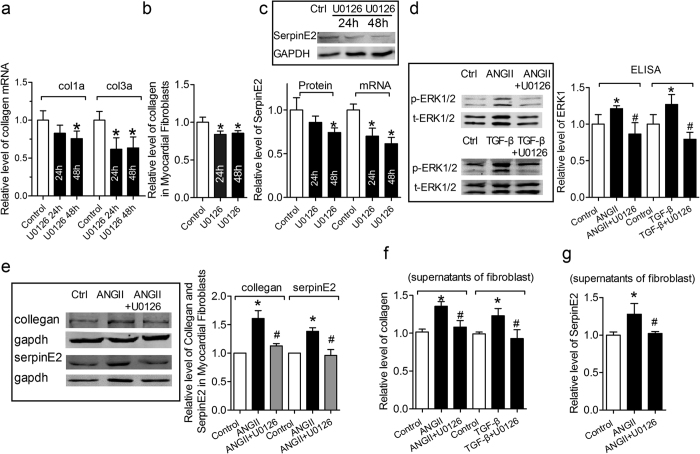
Potential involvement of ERK 1/2 signal pathway in regulating serpinE2 and collagen deposition. (**a**) Collagen mRNA levels measured by qRT-PCR after 24 and 48 h treatment with U0126 (The inhibitor of ERK 1/2 signal pathway). *n* = 10, *P < 0.05 vs. control. (**b**) Collagen expression levels detected by Sircol™ Collagen Assay after 24 and 48 h treatment with U0126. *n* = 6, *P < 0.05 vs. control. (**c**) After treatment with U0126, the protein and mRNA level of serpinE2 was detected in cardiac fibroblasts. *n* = 6, *P < 0.05 vs. control. Full-length blots/gels are presented in Supplementary Figure 8. (**d**) Effect of U0126 on expression of phosphor-ERK1/2 (p-ERK1/2) and total ERK1/2 (t-ERK1/2) in myocardial fibroblasts inducd by AngII or TGF-β. *n* = 8, *P < 0.05 vs. control, ^#^*P* < 0.05 vs. AngII or TGF-β. Left, western blot. Right, Elisa assay. Full-length blots/gels are presented in Supplementary Figure 9 and 10. (**e**) Effect of U0126 on expression of serpinE2 and collagen in myocardial fibroblasts inducd by ANG-II. *n* = 4, *P < 0.05 vs. control, ^#^*P* < 0.05 vs. ANG-II. Full-length blots/gels are presented in Supplementary Figure 11 and 12. (**f**) Effect of U0126 on the collagen content inducd by AngII or TGF-β in supernatants of fibroblasts. *n* = 6, *P < 0.05 vs. control, ^#^*P* < 0.05 vs. AngII or TGF-β. (**g**) Effect of U0126 on expression of serpinE2 inducd by AngII in supernatants of fibroblasts. *n* = 6, *P < 0.05 vs. control, ^#^*P* < 0.05 vs. ANG-II.

ERK1/2 is one of members of the mitogen-activated protein kinase (MAPK) cascade and regulates cell proliferation and differentiation, which is involved in cardiomyocyte hypertrophic growth and fibrosis^24^. The corresponding results of collagen mRNA and content of collagen expression were also reduced after treatment with ERK1/2 inhibitor U0126 (Fig. 6a,b). Published evidence indicates that activation of ERK signaling up-regulates the serpinE2 expression in mouse embryonic fibroblasts^25^. Therefore, ERK1/2 signal pathway in regulating serpinE2 in myocardial fibroblasts is highly expected. To test this hypothesis, the expression of serpinE2 were evaluated and the results showed that, along with the inhibition of ERK1/2 signal pathway by U0126, the protein and mRNA level of serpinE2 were both down-regulated significantly (Fig. 6c).

AngII promotes TGF-β expression and provokes activation of ERK1/2 in atrial and cardiac fibroblasts, leading to ECM accumulation^26^,^27^. In this regard, the potential involvement of ERK1/2 signaling was tested in western blot and Elisa assay. It was showed that Phospho-ERK1/2 expression was significantly up-regulated at 48 h after treatment with AngII or TGF-β1, which could inhibited by ERK1/2 inhibitor U0126 (Fig. 6d). Next, the expression of serpinE2 and collagen were also detected in CFs treated with AngII + U126 or TGF-β1 + U0126 (Fig. 6e). The expression of serpinE2 and collagen were significantly inhibited by pretreatment with U126 compared with AngII or TGF-β1 group in supernatants of CFs (Fig. 6f,g).

### ERK1/2 signal pathway via Elk1 in regulating serpinE2

**Figure 7:**
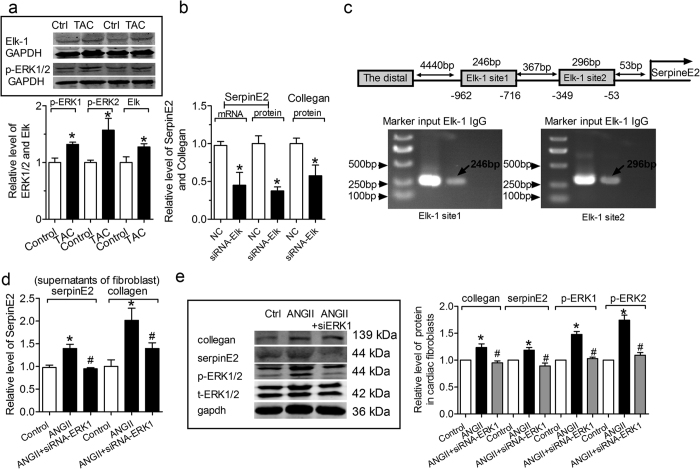
ERK1/2 signal pathway via Elk1 in regulating serpinE2. (**a**) The change of p-ERK1/2 and Elk1 in TAC mouse heart. *n* = 6, *P < 0.05 vs. control. Full-length blots/gels are presented in Supplementary Figure 13 and 14. (**b**) The mRNA level of serpinE2 was decreased by transfection with siRNA Elk in CFs; The level of serpinE2 was decreased by transfection with siRNA Elk in the supernatants of fibroblast; The level of collagen was decreased by transfection with siRNA Elk in the supernatants of fibroblast. n = 6, *P < 0.05 vs. control. (**c**) ChIP testing *in vivo* binding of Elk1 to promoter of Rattus norvegicus genes of serpin family E member 2 (SerpinE2). Top: Schematic representation of the two upstream region of the serpinE2. Bottom: PCR products of Elk1-binding sites following immunoprecipitation with anti- Elk1 antibody. The anti-IgG antibody and H_2_O treatment were used as negative control. The anti- Elk1 antibody was used to target specific immunoprecipitation. ChIP analysis of Elk1 binding to the promoter between-962 and-716 (PCR products 246 bp). Elk1 binding to target Elk1 site 1 activates serpinE2 promoter activity. ChIP analysis of *in vivo* Elk1 site2 binding to the promoter between -349 and -53 bp (PCR products 296 bp). ChIP assay showed that Elk1 binding to this target Elk1 site 2 also did activate serpinE2 promoter activity. (**d**) Effect of knockdown of ERK1 on serpinE2 and the collagen content in supernatants of fibroblasts inducd by ANG-II. *n* = 6, *P < 0.05 vs. control, ^#^*P* < 0.05 vs. ANG-II. (**e**) Effect of knockdown of ERK1 on serpinE2, collagen and p-ERK1/2 in cardiac fibroblasts inducd by ANG-II. *n* = 6, *P < 0.05 vs. control, ^#^*P* < 0.05 vs. ANG-II. Full-length blots/gels are presented in Supplementary Figure 15.

Phosphorylation of ERK1/2 was translocated to the nucleus and activates transcription factors such as Elk1, NF-κB, c-fos and GATA4 etc^28^. In our study, ERK1/2 were activated by TAC and Elk1 was also significantly up-regulated in TAC heart (*P *< 0.05) (Fig. 7a). Among these transcription factors, Elk1 has a close relationship with collagen synthesis^29^,^30^. In our results, knockdown of Elk1 significantly reduced mRNA expression of serpinE2 and the content of serpinE2 in the supernatants of fibroblast (Fig. 7b), suggesting the participation of p-ERK1/2 in ANG-II-induced serpinE2 expression via ERK-dependent transcription of Elk1, which was supported by the notion that the level of collagen was also decreased by transfection with siRNA Elk1 in the supernatants of fibroblast (Fig. 7b). We then subsequently conducted ChIP to further verify the protein-DNA interactions that occur inside the nucleus of CFs cells. More important was that there are 2 optimal and conserved Elk1 binding sites in the promoter region of the serpinE2 gene. The data in Fig. 7c, suggest that Elk1 is able to bind to the serpinE2 promoter, and the binding site located in −962 to −716 bp and −349 to −53 bp upstream of serpinE2 promoter (Fig. 7c). SerpinE2 expression and the collagen was selectively knocked down by siRNA-ERK1 transfection in supernatants of CFs induced by AngII (Fig. 7d). Transfection of siRNA-ERK1 caused an obviously reducdion of ERK1 in CFs (Fig. 7e). Transfection of siRNA-ERK1 prevents the AngII-induced up-regulation expression of serpinE2, collagen and p-ERK level. These results suggested that ERK1/2 signal pathway regulated serpinE2 via Elk1 transcription activator.

## Discussion

The major finding of this work is to clarify the connection of serpinE2/protease nexin-1 with collagen deposition. There results have demonstrated that for the first time the serpinE2/protease nexin-1was up-regulated in fibrosis model both *in vivo* and *in vitro* and the collagen content was significantly reduced by knockdown of serpinE2/protease nexin-1.

In this study, we provide a first direct evidence to prove a close relationship between the different level of serpinE2 and collagen deposition. Using the cardiac fibrosis mouse model at 4 weeks after surgical transverse aortic constriction (TAC)^31^, serpinE2 expression was increased obviously. Additionally, the protein and mRNA level of serpinE2 expression were also dramatically up-regulated *in vitro* induced by AngII or TGF-β stimulation. Moreover, by using serpinE2 shRNA, serpinE2 expression and collagen content were both reduced. In stark contrast, the collagen accumulation in supernatants of fibroblast was observed by exposing myocardial fibroblasts with exogenous serpinE2. Our results showed that serpinE2 increase in collagen deposition and probably is a key player contribute to cardiac fibrosis.

Although the mechanism underlying the contribution of serpinE2 in cardiac fibrosis may not be fully established yet, the relationship of serpinE2 and cardiac fibrosis is likely to be explained upon the following two theories. Firstly, as a Serine protease inhibitor, serpinE2/protease nexin-1 is found in many organs^32^, and it can be secreted into the extracellular space, and next expresses in cytosol and plasma membrane, according to the subcellular localization database (compartments). SerpinE2 can bind to the extracellular matrix on the surface of fibroblasts and several other cultured cells^6^. SerpinE2 forms complexes with certain serine proteases, like urokinase-type plasminogen activator (uPA)^12^, tissue-type plasminogen activator (t-PA)^13^, plasmin and trypsin^14^ in the extracellular environment. Since uPA-PN-1 forms a complex with uPAR (uPA-uPAR-PN-1)^33^, which then binds to the cells and are rapidly internalized and degraded by the low density lipoprotein-related receptor protein (LRP)^5^. uPA play an important role in promoting extracellular matrix (ECM) deposition^34^. Intriguingly, serpinE2 requires to internalize uPAR-bound uPA to form the complex, then further inhibits the uPA that plays a pivotal role by mediating the degradation of extracellular matrix proteins^35^. Secondly, serpinE2 is the phylogenetically closest relative of Plasminogen activator inhibitor type 1 (PAI-1)^15^ that is implicated in the pathology of fibrosis in multiple organs including the heart, lung, kidney, liver and skin^16^ SerpinE2 is an inhibitor of uPA and tissue plasminogen activator but has a larger inhibition spectrum than PAI-1, and it may also modulate extracellular matrix degradation in vascular cell^10^. SerpinE2 is thought to have a pathogenic role in the development of another fibrotic disease, and scleroderma^36^.

**Figure 8:**
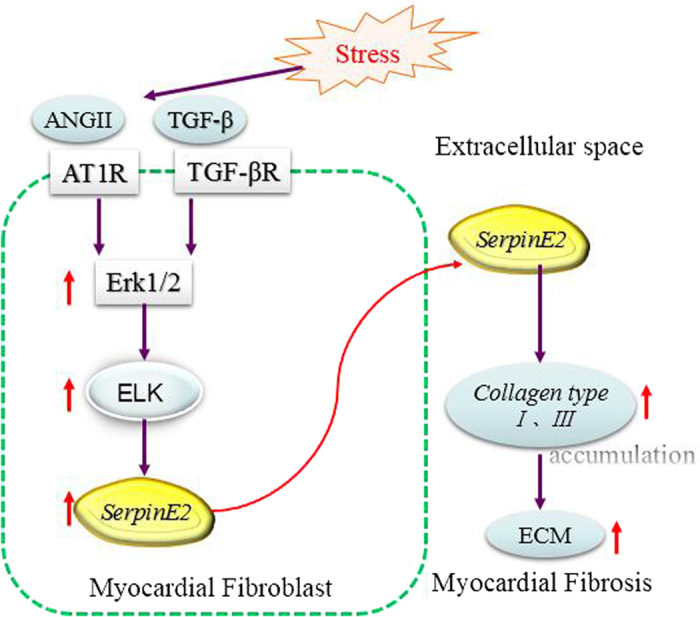
Model demonstrating possible molecular basis of SerpinE2 induced collagen deposition in myocardial fibrosis. Pressure overload can trigger some response: activation of renin-angiotensin-aldosterone system, in particular, systemic and local production of Ang II. Elevated AngII and TGF-β promote fibrotic responses of the heart, and then activates MAPK-ERK1/2 signaling. Phospho-ERK1/2 can further activate ERK-dependent transcription activator Elk1 in myocardial fibroblasts which in turn activates SerpinE2.

Myocardial fibrosis is a major player in cardiac remodeling that is an important pathophysiological process along with the proliferation of cardiac fibroblasts and excessive deposition of extracellular matrix between musclar fibers^1^,^2^. The published evidence has shown that several mediators are invloved in cardiac fibrosis^37^, such as the renin/angiotensin/aldosterone system, inflammatory cytokines chemokines, reactive oxygen species, endothelin-1, and growth factors TGF-β etc. Elevated AngII is associated with the fibrosis in the heart^38^, and the stimulation of AngII type 1 receptor (AT_1_R) activates ERK1/2 by uncoupling G protein-dependent and β-arrestin2-dependent pathways^39^, by which ERK1/2 can further activate ERK-dependent transcriptional responsiveness of Elk1, GATA4, and the ANP factor promoter^40^. Our study showed that AngII and transforming growth factor TGF-β promote fibrotic responses of the heart^41^ and induce fibrosis, at meantime, both factors may activates Smad and MAPK-ERK1/2 in myocardial fibroblasts via transcription factors-Elk1 which in turn activates serpinE2. SerpinE2 expression is thus up-regulated *in vitro* and *in vivo* and inhibits proteolysis like uPA and prevents collagen degradation (Fig. 8). uPA/uPAR system plays crucial roles in ECM deposition^34^, which is associated with myocardial fibrosis and remodeling^42^.

This observation has clearly indicated that serpinE2 is elevated with accumulation of collagen and the most importantly, suggesting that the laboratory examination of serum level of serpinE2 would be a measure to predict cardiac fibrosis and serpinE2 could be served an important diagnostic profibrotic marker of cardiac fibrosis. In addtion, easy operation of plasma detection using Elisa kit and less plasma sample required (0.01–0.1 ml) to test serpinE2 are also advantages in clinical practise. The early myocardial fibrosis is barely detected by MRI and the biopsy is a restricted a invasive diagnostic testing, heretofore, it is very prospective to diagnose myocardial fibrosis in early stage and then puts serpinE2 plasma detection in an very important position.

This novel finding provides evidence demonstrating that serpinE2 is one of the molecular basis of cardiac fibrosis. Stress induced the ERK1/2 signaling may promote the secretion of serpinE2 from myocardial fibroblasts leading to the accumulation of extracellular matrix protein and contribution to cardiac fibrosis.

## Materials and Methods

### Ethics statement

The study was approved by the Institutional Animal Care and Use Committee of Harbin Medical University, P.R. China (No. HMUIRB-2008-06). All experimental procedures were performed in accordance with the Guide for the Care and Use of Laboratory Animals, published by the US National Institutes of Health (NIH Publication No. 85–23, revised 1996).

### Preparation of pressure overload–induced cardiac fibrosis mouse model

Adult male C57BL/6 mice from the Second Affiliated Hospital of Harbin Medical University, (Harbin, China) were subjected to pressure overload by surgical transverse aortic constriction (TAC) to induce cardiac fibrosis as described 17,18. The mice about 22–25 g weight were subjected to TAC surgery under anesthesia with 1% pentobarbital sodium intraperitonelly injection and kept at animal facility for another 4 weeks to sucessully develop cardiac fibrosis. All protocols and procedures for animal uses was pre-approved by the Institutional Animal Care and Use Committee of Harbin Medical University, P.R. China.

### Masson staining

The left ventricular tissues of mouse hearts of normal myocardium and TAC model were obtained immediately after the mouse were sacrificed and fixed in 4% paraformaldehyde solution and Masson’s trichrome staining was performed 24 h after that to detect the fibrotic areas with blue compared with myocardial tissue with red staining under microscope examination. The extent of cardiac fibrosis in the heart of TAC mouse was assessed by calculating collagen volume fraction. All quantitative evaluations were carried out by Image-Pro Plus software (version 6.0).

### Isolation and culture of cardiac fibroblasts

Primary cardiac fibroblasts were isolated from 1 to 3 day-old neonatal rat with 0.25% Trypsin-EDTA (Gibco, Grand Island, NY) as previously described 19. Cardiac fibroblasts were collected by discarding the cloudy medium containing cardiomyocytes through selective adhesion of non-myocytes at a 1.5 h pre-plating interval. The isolated cells were then incubated in DMEM with 10% fetal bovine serum (BI Company, Israel) and cultured at 37 °C in 5% CO_2_ and 95% air. CFs at the 3rd or 4th passages were used in our experiments. After starvation in serum-free medium for 24 h, 50 nM AngII (Sigma–Aldrich, St. Louis, MO, USA), or 10 ng/ml recombinant human TGF-β1 protein (Sino Biological Inc., China), or 10 ng/ml recombinant human serpinE2 (Proteintech Inc., USA) were applied to cardiac fibroblasts, respectively, for 24 h and 48 h.

### Transfection shRNA SerpinE2, siRNA ERK-1 or siRNA Elk1

For transfection, cardiac fibroblasts were cultured with serum-free medium once and then incubated with serum-free medium for 6 h. The shRNA serpinE2 (PN-1 shRNA Plasmid (h): sc-45254-SH Santa Cruz Biotechnology Inc., USA), siRNA ERK-1 and siRNA Elk1 (Guangzhou RiboBio Co. Ltd., China), and X-treme GENE siRNA Transfection Reagent (Roche, USA) were mixed, respectively, with Opti-MEM^®^ Reduced Serum Medium (Gibco, Grand Island, NY), and then cardiac fibroblast were treated with each mix for transfection, according to the manufacturer’s protocol.

### Knockdown SerpinE2 *in Vivo*

SerpinE2-RNAi-lentivirus were purchased from Shanghai GeneChem Company (Shanghai, China). The SerpinE2-RNAi #1 sequence was 5-TTGGCATTACTGAGATGTT-3 and the shRNA Negtive control sequence was 5-TTCTCCGAACGTGTCACGT-3. Groups of 10 mice (C57BL/6; Taconic Laboratories) were injected via the tail vein with 1*10 ^7^TU of either commercial serpinE2-RNAi- lentivirus (LV-RNAi) or Negtive control lentivirus (LV-NC). One week after injection, these mice were subjected to pressure overload by TAC four weeks.

### Chromatin immunoprecipitation assay (ChIp)

Primary cardiac fibroblasts were cultured as described above. Chromatin immunoprecipitation assay was performed with the ChIP assay kit (catalog #26156; Pierce Thermo Fisher Scientific) according to the manufacturer’s instructions. ChIP testing *in vivo* binding of Elk1 to promoter of Rattus norvegicus genes of serpin family E member 2 (SerpinE2, NM_019197.1). The PCR primers (Elk1 site1 ChIP Forward primer 1: 5′-GGGTGGAGGAGTGTCTGGCATG-3′, Reverse primer 1: 5′-GTCGCCTGGGTGTCCCTTTCTGC-3′. Elk1 site2 ChIP F2: 5′-CTTCGGTGTCGCCCACTCCTCTC-3′, R2:5′-GTCGTGCCGCCCTCGTTGCCG-3′) were designed to amplify Elk1 site1 (246 bp) and Elk1 site2 (296 bp) fragments, respectively, from selected genomic regions, respectively. RT-PCR of genomic regions containing the putative Elk1-binding sites was performed in triplicate. Amplification of the distal as negative control, a 181 bp fragment spanning selected genomic regions, was performed with oligos 5′-TTCCCTCAGAACAATAACGCAG-3′ and 5′-CCTTCCAAGTAGAAGCTTGGAATG-3′.

### Western blotting

Cardiac tissues or cardiac fibroblasts were also lysed with standard sample buffer. After boiling the samples for 10 min, the protein samples were fractionated by 10% SDS–PAGE. Primary antibody against SERPINE2 (Rabbit polyclonal, ab75348) was purchased from Abcam (Cambridge, MA); and antibody against p-ERK1/2 and Elk1 were purchased from Santa Cruz Biotechnology (Santa Cruz, CA). Western blot bands were quantified using Odyssey v1.2 software by measuring the band intensity for each group and normalizing to GAPDH (anti-GAPDH antibody from Kangcheng, Shanghai, China) as an internal control.

### RNA isolation and quantitative real-time RT-PCR (qRT-PCR)

Total RNA were extracted from plasma or cardiac tissues from TAC mice. Total RNA was isolated from 1 ml plasma using phenol/chloroform extraction procedures. For qRT-PCR, the cDNA was assessed with 5X All-In-One RT MasterMix (abmGood, Canada). The EvaGreen qPCR Mastermix Kit (abmGood, Canada) was used in real-time PCR for relative quantification. Collagen, type I alpha1, Collagen type III alpha1, and serpinE2 mRNA levels were quantified by fast real-time PCR system (ABI 7500, Applied Biosystems, Carlsbad, CA, USA), and GAPDH was set as an internal control. The 2^−ΔΔCt^ method was applied for the data analysis and the data were normalized and converted into relative mRNA expression.

### Extract samples from cardiac fibroblasts and supernatant of fibroblasts

The culture supernatant was collected in EP tubes and centrifuged at 3000 rpm for 20 min, and then extracted the supernatant into another tagged EP tubes for ELISA or Sircol soluble collagen assay. After that, the cardiac fibroblasts were washed with PBS solution for 3 times and collected with RIPA-PI mixture (100:1) (Thermo Scientific, USA) to make cells lysis solution. The cells lysis solution was centrifuged at 13500 rpm for 15 min.

### ELISA

The levels of SerpineE2 in plasma, culture media (supernatant of fibroblasts), or tissue homogenates from the culture media (Cell culture supernatant) or Plasma or tissue homogenates were measured by using the ELISA kits (EL- R2534c/ M2646c) and Extracellular Signal-Regulated Kinase (ERK) ELISA Kit (E-EL-M0480c) (Elabscience Biotechnology, Wuhan, China), according to the manufacturer’s instructions.

### Quantification of collagen

Total collagen content was determined by using Sircol soluble collagen assay (Biocolor, Belfast, UK) according to the manufacturer’s instructions. 200 μl aliquots of the alkali dye solutions was collected from each group and added to 96-well plates for the absorbance using a microplate reader (SUNRISE, Switzerland) at 555 nm. The absorbance was directly proportional to the amount of newly formed collagen in the cell culture supernatant.

### Immunofluorescence

Cardiac fibroblasts were washed briefly with cold PBS for 3 times and fixed with 4% paraformaldehyde for 15 min and then penetrated by Triton X-100 (Sigma-Aldrich, St. Louis, MO). The cells were incubated with primary antibody against SERPINE2 (Rabbit polyclonal, Abcam Cambridge, MA) or Vimentin (Mouse polyclonal, Abcam Cambridge, MA) or Troponin I (Rabbit polyclonal, Abcam Cambridge, MA) overnight at 4 °C. Nuclei were stained with DAPI (Roche Molecular Biochemicals) for 5 min at room temperature, and immunofluorescence was analyzed under a fluorescence microscope (Nikon 80i, Japan).

### Data Analysis

Averaged data were expressed as mean ± SEM. Two-group–only comparisons were performed by paired Student’s t-test. Multiple-group comparisons for Collagen content, ELISA real-time RT-PCR and Western blot experiments were analyzed with 1-way ANOVA followed by Bonferroni’s post hoc tests. the P value less than 0.05 was considered as the significant difference.

## Additional Information

**How to cite this article**: Li, X. *et al*. Overexpression of SerpinE2/protease nexin-1 Contribute to Pathological Cardiac Fibrosis via increasing Collagen Deposition. *Sci. Rep.* **6**, 37635; doi: 10.1038/srep37635 (2016).

**Publisher’s note:** Springer Nature remains neutral with regard to jurisdictional claims in published maps and institutional affiliations.

## Supplementary Material

Supplementary Figure
