# Supplementary material for: Overexpression of SerpinE2/protease nexin-1 Contribute to Pathological Cardiac Fibrosis via increasing Collagen Deposition
Source: Sci Rep. 2016 Nov 23;6:37635. doi: 10.1038/srep37635 (PMC5120308; doi:10.1038/srep37635)
Supplement: Supplementary Figure [file srep37635-s1.pdf]

# Overexpression of SerpinE2/protease nexin-1 Contribute to Pathological Cardiac Fibrosis via increasing Collagen Deposition

Xuelian Li [1]*, Dandan Zhao [1]*, Zhenfeng Guo[1,2]*, Tianshi Li[1], Muge Qili [1], Bozhi Xu[1], Ming Qian[1], Haihai Liang[1], Xiaoqiang E[3], Samuel chege Gitau[1,4], Lu Wang[1], Longtao Huangfu[1], Qiuxia Wu[1], Chaoqian Xu[1], Hongli Shan[1]✉.

Supplementary Figure 1

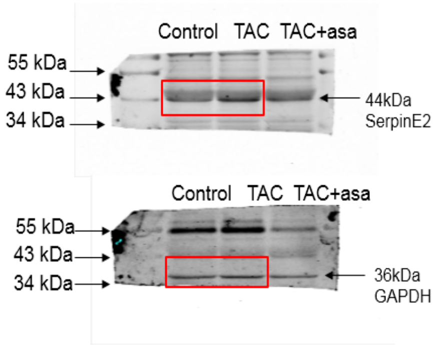


Supplementary Figure 1.

The full-length blots/gels is the display of cropped gels and blots from Fig 1.e.

Supplementary Figure 2

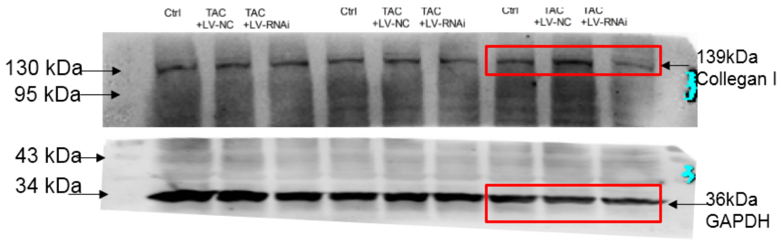


Supplementary Figure 2. The full-length blots/gels is the display of cropped gels and blots from Fig 1.i.

Supplementary Figure 3

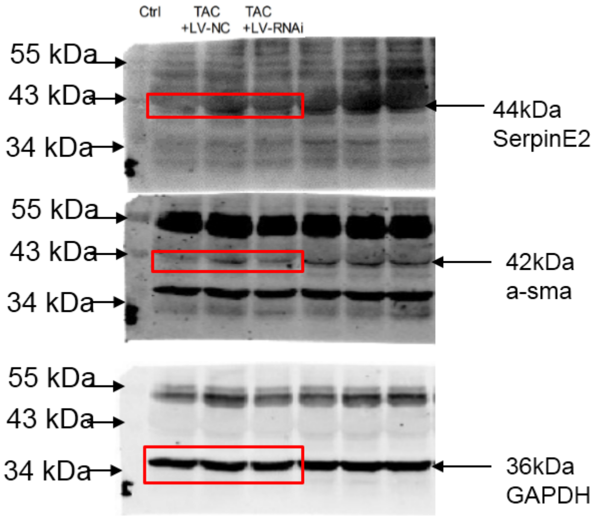


Supplementary Figure 3

The full-length blots/gels is the display of cropped gels and blots from Fig 1.i.

Supplementary Figure 4

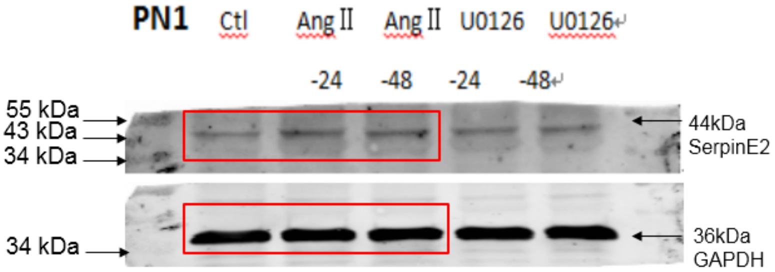


Supplementary Figure 4

The full-length blots/gels is the display of cropped gels and blots from Fig 2.d.

Supplementary Figure 5

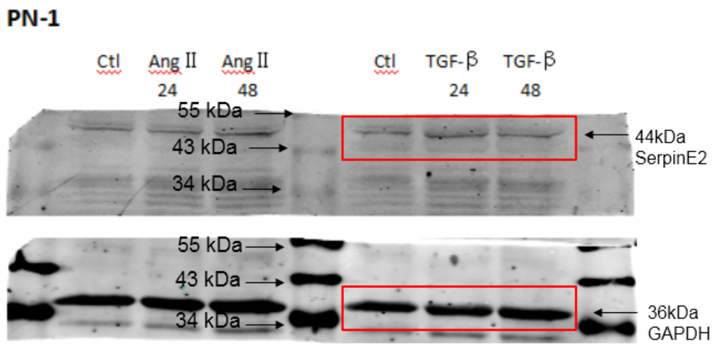


Supplementary Figure 5

The full-length blots/gels is the display of cropped gels and blots from Fig 3.d.

Supplementary Figure 6

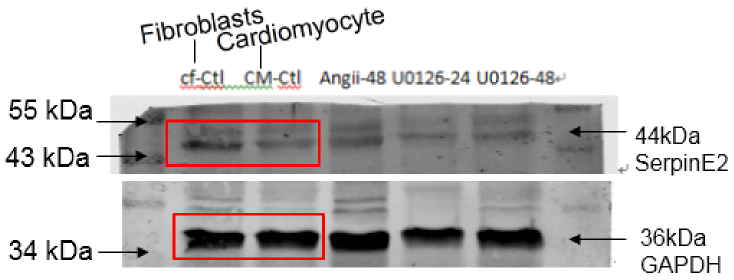


Supplementary Figure 6

The full-length blots/gels is the display of cropped gels and blots from Fig 4.a.

Supplementary Figure 7

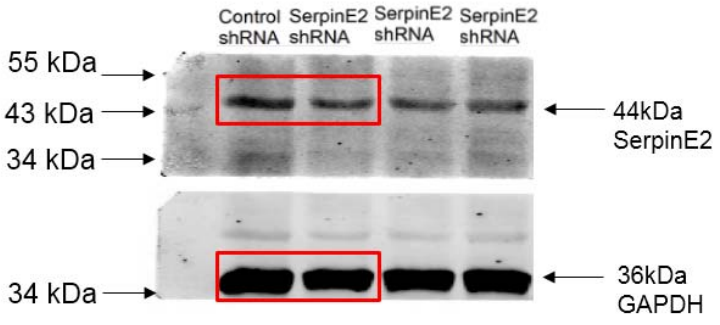


Supplementary Figure 7

The full-length blots/gels is the display of cropped gels and blots from Fig 5.a.

Supplementary Figure 8

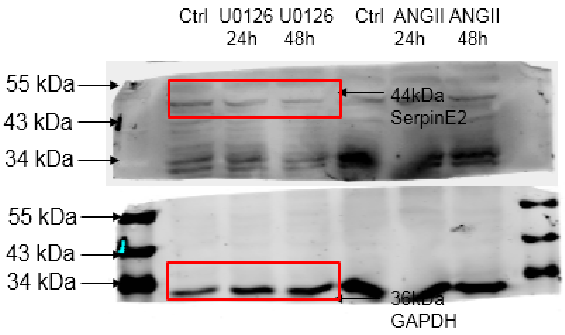


Supplementary Figure 8

The full-length blots/gels is the display of cropped gels and blots from Fig 6.c.

Supplementary Figure 9

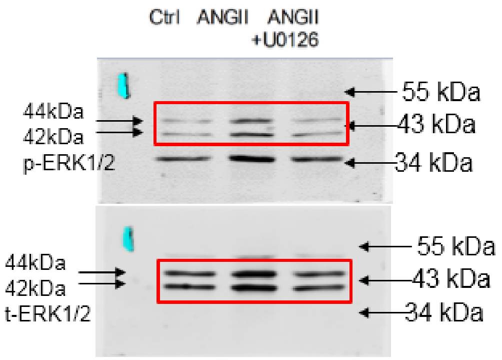


Supplementary Figure 9

The full-length blots/gels is the display of cropped gels and blots from Fig 6.d.

Supplementary Figure 10

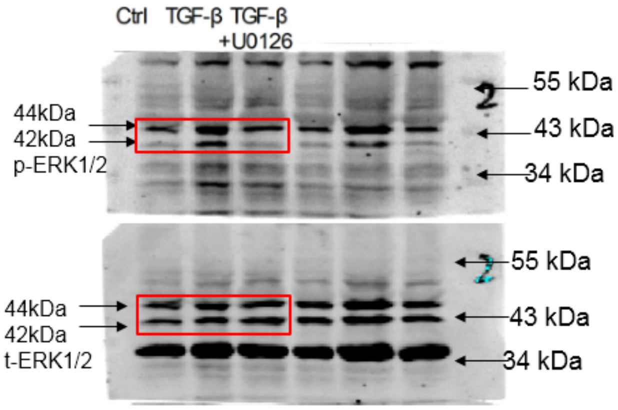


Supplementary Figure 10

The full-length blots/gels is the display of cropped gels and blots from Fig 6.d.

Supplementary Figure 11

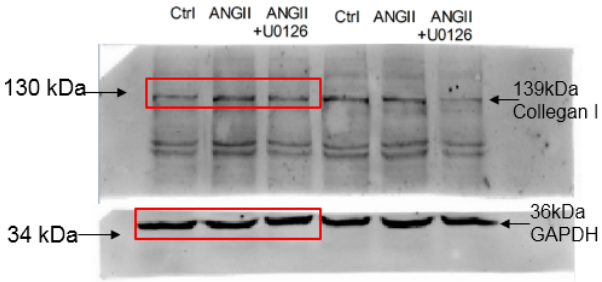


Supplementary Figure 11

The full-length blots/gels is the display of cropped gels and blots from Fig 6.e.

Supplementary Figure 12

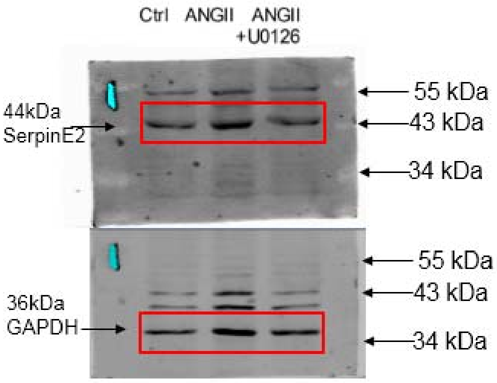


Supplementary Figure 12

The full-length blots/gels is the display of cropped gels and blots from Fig 6.e.

Supplementary Figure 13

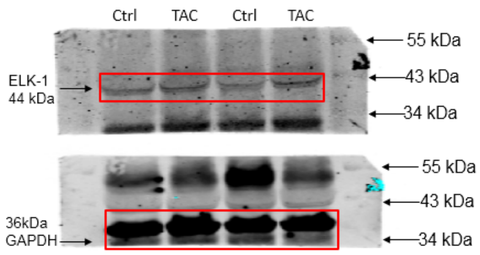


Supplementary Figure 13

The full-length blots/gels is the display of cropped gels and blots from Fig 7.a.

Supplementary Figure 14

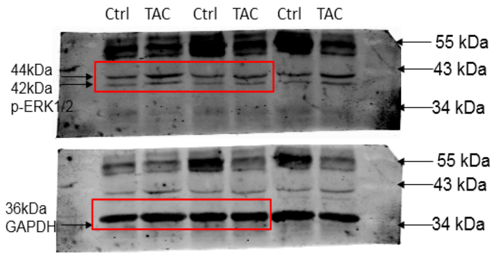


Supplementary Figure 14

The full-length blots/gels is the display of cropped gels and blots from Fig 7.a.

Supplementary Figure 15

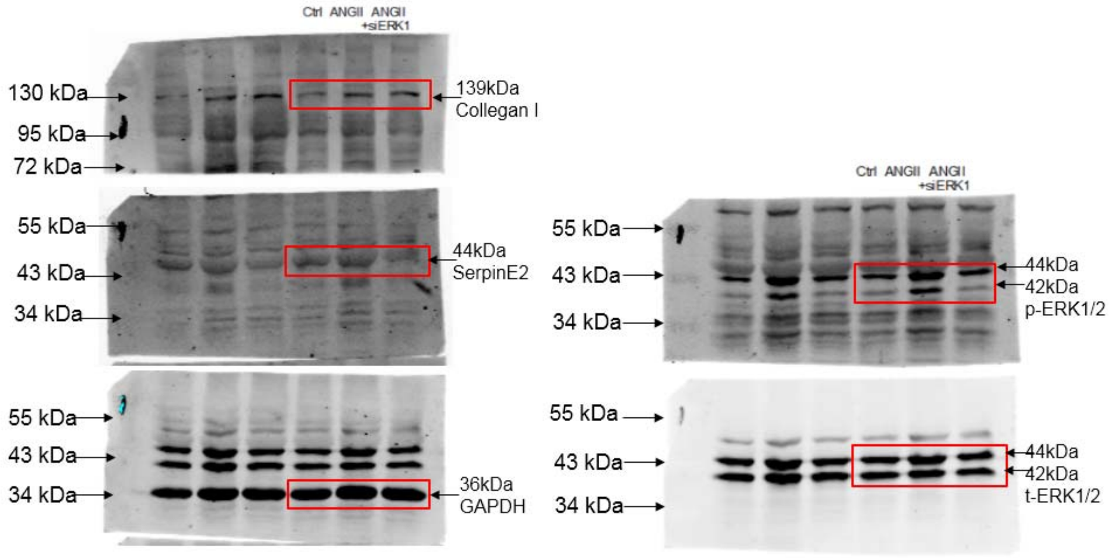


Supplementary Figure 15

The full-length blots/gels is the display of cropped gels and blots from Fig 7.e.

.
